# Laser-Self-Mixing Interferometry for Mechatronics Applications

**DOI:** 10.3390/s90503527

**Published:** 2009-05-12

**Authors:** Simona Ottonelli, Maurizio Dabbicco, Francesco De Lucia, Michela di Vietro, Gaetano Scamarcio

**Affiliations:** Dipartimento Interateneo di Fisica “M. Merlin”, Università di Bari, and CNR-INFM Laboratorio Regionale “LIT3”, Via Amendola 173, I-70126, Bari, Italy; E-Mails: dabbicco@fisica.uniba.it; delucia@fisica.uniba.it; divietro@fisica.uniba.it; scamarcio@fisica.uniba.it

**Keywords:** physical sensors, displacement measurement, self-mixing, interferometry, mechatronics, semiconductor laser, six degrees-of-freedom

## Abstract

We report on the development of an all-interferometric optomechatronic sensor for the detection of multi-degrees-of-freedom displacements of a remote target. The prototype system exploits the self-mixing technique and consists only of a laser head, equipped with six laser sources, and a suitably designed reflective target. The feasibility of the system was validated experimentally for both single or multi-degrees-of-freedom measurements, thus demonstrating a simple and inexpensive alternative to costly and bulky existing systems.

## 1. Introduction

A common task in precision manufacturing and micromachining is the real-time control of the tool centre point (TCP) motion and positioning. In a single-axis coordinate measuring machine (CMM), the TCP moves along linear guideways and its displacement is monitored by means of mechanical or optical encoders. However, even if a slide moves linearly in one direction, which we will assume to be the x-axis, the generation of motion in the other five Degrees-of-Freedom (DOFs) is unavoidable because of geometric defects of guideways, thermal expansion and/or other mechanical imperfections. For this reason, six degrees of kinematic freedom – three translational (linear motion, straightness and flatness) and three rotational (yaw, pitch and roll) – are required for the full description of the position and orientation of the TCP, although the dynamic ranges and required precision may largely vary for each DOF.

The last decade has seen an increasing interest in the development of sophisticated devices capable of sensing the six DOFs motion of a rigid body. Laser interferometry is one of the most wide distributed and high performing phase sensitive techniques for length-related metrological measurements, precision engineering and industrial applications, as well as for advanced scientific applications. Laser calibration systems based on interferometric techniques are the most appropriate instruments for measurement, not only of linear displacement over long distances, but also of straightness, pitch, yaw and roll rotations [1-4]. Interferometric sensors are usually capable of high resolution, fast response over a large measurement range and great reliability. However, the great number of optical components and the critical procedure of optical path alignment result in bulky instrumentation and high costs. The replication of a single interferometric set-up for the simultaneous evaluation of more than one DOF can thereby be problematic and often inconvenient.

In fact, current trends show an increasing number of positioning systems based on the integration of multiple interferometers with other sensing techniques. Common, for example, is the evaluation of linear displacement by means of an interferometric technique, and transverse displacements by means of position sensitive detectors, like CCDs or quadrant photodetectors (QD) [5-8]. This “hybrid” approach often ensures high measurement accuracy, but does not solve the issue of the complexity and the high cost of the experimental arrangement, since the sensor requires several optical elements for the separation, the deviation and the polarization of the laser beams. Up to now, no commercial all-interferometric system for the assessment of the six DOFs motion is available.

An innovative approach is represented by Laser - Self - Mixing (LSM) interferometry, which does not require any reference arms or external detectors and it is conveniently replicable because of a robust and almost self-aligned setup, an intrinsic resolution more than adequate for most industrial applications, and a simple and single-channelled reading electronics. Although the LSM has been widely studied and exploited for metrological applications since more than three decades, it has rarely been applied to the simultaneous measurements of more than one DOF.

Recently, the authors reported on a compact interferometric system based on the LSM effect, capable of tracking the longitudinal displacement of a target with sub-micron resolution up to 1 meter simultaneously with yaw and pitch rotations up to ± 0.45° [9], and with straightness and flatness displacements up to ± 1 mm [10].

In this paper, we extend the measurement technique to the evaluation of the roll angle up to ± 0.45°, and we finally present an integrated all-interferometric sensor totally based on the LSM effect for the measurement of six DOFs of a moving target. The described sensor is composed of six laser sources and a suitably designed reflective target, formed by three reciprocally tilted mirrors. The measurement technique is based on the differential measurement of linear displacement by pairs of identical self-mixing interferometers (SMIs), each formed only by a laser diode package with integrated monitor photodiode, a collimated lens, and a reflective surface orthogonal to the optical axis.

**Figure 1.**
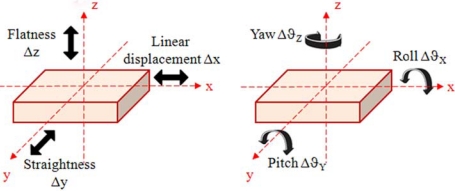
Coordinate system convention, linear (at the left) and angular (at the right).

Throughout the manuscript, the coordinate convention illustrated in Figure 1 will be adopted:

- the x-axis is the direction of linear motion and any displacement along this axis will be indicated by Δx;
- the y-axis is the direction of the straightness displacement indicated by Δy;
- the z-axis is the direction of the flatness displacement indicated by Δz;
- roll is the angular motion around the x-axis and any rotation around this axis will be indicated by Δϑ_x_;
- pitch is the angular motion around the y-axis and any rotation around this axis will be indicated by Δϑ_y_;
- yaw is the angular motion around the z-axis and any rotation around this axis will be indicated by Δϑ_z_.

The paper is organized as follows: the self-mixing interference principle is introduced in Section 2. The measurement technique for two rotations (yaw and pitch) is reported in Section 3. The extension of the basic principle to the assessment of transverse DOFs is described in Section 4. Considerations about the performance and the integration of the system for the simultaneous measurement of more DOFs are discussed in Section 5 and 6, respectively. Finally, conclusions are drawn in Section 7.

## 2. Laser-Self-Mixing Interference

### 2.1. Basic principle

**Figure 2.**
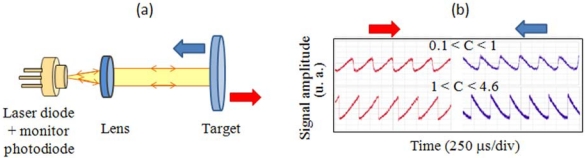
(a) Schematics of the LSM interferometer, with the target moving forward (red arrow) or backward (blue arrow) with respect to the laser source. (b) Experimental oscilloscope waveforms obtained for different values of the feedback parameter C in presence of a linear continuous displacement of the target at a speed of 1 mm/s in backward (blue traces) and forward (red traces) direction with respect to the laser source.

The LSM interference (see Figure 2) takes place when a portion of the light emitted by a laser source is reflected back into the laser cavity by an external reflector, such as a mirror or a diffusive target, and interferes with the standing wave inside the laser cavity, causing an amplitude and frequency modulation of the output laser beam [11-12].

Several benefits derive from the LSM scheme. First, the optical alignment procedure is not so critical as in external interferometers, since there is only one measurement arm, the reference arm provided by the laser itself. Second, the reference arm is self-stabilized once the driving current and the heat sink temperature of the laser source are kept constant. Third, the output signal can be detected by a photodiode placed anywhere along the optical path. The geometry of the setup can be optimized if the signal is detected by means of the photodiode integrated into most commercial laser packages for power monitoring, which allows the laser to be used as source and detector at the same time. The basic setup, sketched in Figure 2(a), is thereby made of only a laser source, a collimating lens, a remote target, and a neutral attenuator if any adjustment of the feedback power is required. Actually, this setup can be considered as an evolution of the Michelson interferometer with the reference arm folded on itself toward the laser source, whose front facet serves as the beam splitter.

Fourth, the feedback regime, i.e. the relative amount of light coupled back into the laser, affects the characteristics of the output signal in a non-linear way, allowing for the identification of the sign of the displacement by means of a single quadrature. A useful classification of the feedback regimes for metrological purposes can be performed by adopting the C-value as selective parameter [13], where C is the feedback parameter [14] defined as follows:

(1) $$\text{C}=ɛ\sqrt{\frac{{\text{R}}_{3}}{{\text{R}}_{2}}}\left(1-{\text{R}}_{2}\right)\sqrt{1+\alpha^{2}}\frac{\text{x}}{l\cdot n_{\text{eff}}}$$

Expression (1) depends on a combination of laser dependent parameters (R_2_ is power reflection coefficient of the front laser facet, *l* is the laser cavity length, n_eff_ is the effective refractive index of the active medium, α is the linewidth enhancement factor) and system adjustable parameters (R_3_ is the power reflection coefficient of the target, also including the power attenuation possibly present in the external cavity, and ε < 1 is a constant referred to as the mode matching factor).

When C ≪ 1 (very weak feedback regime), the output waveform is represented by a sine function as in classical interferometry. In this regime the laser behavior is \\quite unperturbed with respect to isolated laser operations, and the symmetry of the waveform requires the duplication of the measurement channel in order to recover the direction of target displacement. With reference to the oscilloscope waveforms produced during a linear displacement of the target and reported in Figure 2(b), when 0.1 < C < 1 (weak feedback regime), the waveform becomes progressively distorted as C approaches unity, with an asymmetry related to the direction of motion of the external reflector, until for 1 < C < 4.6 (moderate feedback regime), the laser becomes bi-stable and the waveform is visibly sawtooth-like, with abrupt switches every time the phase changes by 2π. Lastly, for C > 4.6 (strong feedback regime, not represented in Figure 2(b), the system becomes multi-stable and the waveform collapses to a very noisy one [15].

### 2.2. Measurement of linear displacements

On the basis of the above classification, operation in the moderate feedback regime represents the most convenient solution for displacement measurements along the longitudinal x-axis, since the module of the displacement can be simply recovered by the count of the total number of produced fringes, whereas the direction of displacement can be recovered via the sign of the sharp transitions of the sawtooth-like signal. In more detail, the standard analysis in the self-mixing configuration consists of the AC signal amplification by a trans-impedance amplifier, followed by the time-derivation of the output signal, which converts the sawtooth-like fringes in a series of positive and negative spikes, whose sign depends on the direction of the motion of the target. Finally, the net number of counts N = N_+_ - N_-_, given by the algebraic sum of the positive (N_+_) /negative (N_-_) counts, returns the linear displacement Δx as:

(2) Δx=N⋅λ/2

where N and λ are the net number of fringes and the wavelength of the laser.

### 2.3. Role of the collimation condition of the laser beam

A great variety of theoretical investigations and experimental improvements have been developed on this subject during the last twenty years. However, almost all of them take into account collimated laser beams [16-20], which, as we are going to demonstrate in the following, impose substantial limitations on applications of LSM to real world environments.

Since a collimated laser beam will bounce back and forth along the external cavity without significant power loss, a reduction of the feedback power in order to satisfy the moderate feedback condition is often needed. Different approaches have already been used, e.g. the insertion of an optical attenuator in the external cavity or the adoption of a weakly diffusive target. However, the solutions adopted so far always introduce a fixed attenuation irrespective of the target distance x. Being C directly proportional to x, the system will necessarily cross the upper threshold C = 4.6 for long enough cavities, thereby becoming unstable and limiting the useful measurement range to the condition x_max_/x_min_ = 4.6. The longest reported linear measurement range, achieved without any change of experimental conditions (optics alignment, feedback attenuation, etc.), is Δx = 1.2 m [21].

At variance to this approach, we exploit an alternative one [22], which consists of the reduction of the feedback power by inserting power losses that are dependent on the external cavity length, thus balancing the linear increase of the feedback parameter C. The easiest way to achieve length-dependent losses consists of using a slight divergent laser beam. In this context, the plane wave approximation is firmly unable to correctly model the operation of LSM displacement sensors when the laser beam is not perfectly collimated. To counter this problem, we exploited the ABCD matrix formalism to develop a simulation model of the laser/mirror interaction in the Gaussian beam approximation.

The aim of this Section is to summarize the main equations of the model. Given the optic axis x, the fundamental Gaussian beam at wavelength λ is completely defined by any one of the following parameters: the beam waist w_0_, that we assume to coincide with the center of laser cavity, the Rayleigh range x_0_ or the beam divergence θ_0_, related by $w_{0}^{2}={\left(\pi \theta_{0}/\lambda \right)}^{-2}=(\lambda x_{0}/\pi )$. Since a semiconductor diode laser usually emits an asymmetric beam with different divergences in the planes parallel and orthogonal to the junction, two of the above parameters, one for each plane, will be necessary to specify the fundamental elliptical Gaussian mode.

The propagation of the Gaussian beam through a linear optical system can be conveniently described by propagating its complex parameter *q*(*x*) = *x* + *j x_0_* according to the *ABCD* law, namely *q_2_* = (*Aq_1_* +*B*)*/* (*Cq_1_* +*D*), where *q*_1_ and *q*_2_ defines the complex Gaussian parameter before and after, respectively, an optical system characterized by the linear 2×2 matrix [(*A, B*),(*C, D*)]. For the elliptical Gaussian beam, the parameter propagation can be defined by the separate consideration of the propagation of two complex parameters, *q_y_*(*x*) and *q_z_*(*x*). With reference to Figure 2(a), where the schematics of the LSM interferometer are presented, the only optical element crossed by the beam is the aspherical lens, whose matrix we take as the simple thin-lens matrix [ (1, 0),(−*f* ^-1^, 1)] with *f* the nominal focal length of the lens. The mirror reflection is accounted for by the identity matrix and its small tilt angles: *θ*_y_ (pitch) around the *y* axis and *θ*_z_ (yaw) around the *z* axis, contribute to displace the backward beam centre off-axis according to *y*_0_ ≈ *xθ*_z_ and *z*_0_ ≈ *xθ*_y_. The complex Gaussian beam parameters of the backward beam at the lens (*q_x_*) and at the original waist position (*q_D_*) are then given by:

(3) $${\text{q}}_{\text{Ly},\text{z}}=\frac{{\text{q}}_{0\text{y},\text{z}}(\text{f}-2\text{x})+\text{df}+2\text{x}(\text{f}-\text{d})}{\text{f}-\text{d}-{\text{q}}_{0\text{y},\text{z}}}$$

and

(4) $${\text{q}}_{\text{Dy},\text{z}}=\frac{{\text{q}}_{0\text{y},\text{z}}[2\text{x}(\text{d}-\text{x})-\text{f}(2\text{d}-\text{x})]-2(\text{d}-\text{f})[\text{d}(\text{f}-\text{x})+\text{fx}]}{2\text{x}(\text{d}-\text{f})-\text{f}(2\text{d}-\text{f})-2(\text{f}-\text{x}){\text{q}}_{0\text{y},\text{z}}}$$

respectively, where *d* is the distance of the lens from the laser. The beam waists can be calculated accordingly, provided that $w_{y,z}^{2}(x)=-\lambda /\pi ℑ[q_{y,z}^{-1}(x)]$.

The important element to consider is that the power lost by a Gaussian beam through any on-axis circular aperture of radius *a* is the *exp[*−2a^2^/(w_y_*(*x*)* w_z_(x*))]* fraction of the incident power. Since a divergent Gaussian beam will grow in size propagating back and forth along the cavity, the longer the cavity, the higher the fractional power loss at any of the finite aperture optical elements, the most important of which are in our case the collimation lens and the diode facet itself (the losses at the mirror are in fact mostly due to the non unitary reflection coefficient *R*_3_). By taking the origin of the coordinate system at the beam waist position and aligning the junction plane of the diode laser with one of the Cartesian planes, the contribution to the feedback power ratio due to the Gaussian beam profile can be calculated as:

(5) $$\frac{{\text{P}}_{\text{F}}}{{\text{P}}_{0}}=\exp\left[-\frac{2{\text{a}}^{2}}{{\text{w}}_{\text{y}}(\text{d}){\text{w}}_{\text{z}}(\text{d})}-\frac{2{\text{a}}^{2}}{{\text{w}}_{\text{y}}(\text{d}+2\text{x}){\text{w}}_{\text{z}}(\text{d}+2\text{x})}-\frac{2{\text{w}}_{0\text{y}}{\text{w}}_{0\text{z}}}{{\text{w}}_{\text{y}}(2\text{d}+2\text{x}){\text{w}}_{\text{z}}(2\text{d}+2\text{x})}\right]$$

and the feedback parameter C in eq. (1) can be re-written as:

(6) $$\text{C}=\sqrt{\frac{{\text{P}}_{\text{F}}}{{\text{P}}_{0}}}\sqrt{\frac{{\text{R}}_{3}}{{\text{R}}_{2}}}\left(1-{\text{R}}_{2}\right)\sqrt{1+\alpha^{2}}\frac{\text{x}}{l\cdot n_{\text{eff}}}$$

**Figure 3.**
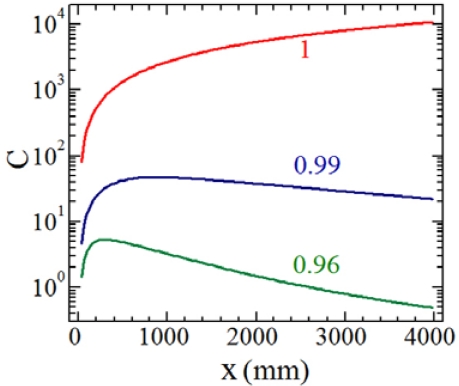
C-parameter for increasing distances x between the laser and the target by varying the ratio d/f, reported near each curve for ease of comparison. All curves have been calculated for typical values of diode laser parameters: w_0x_ = 8°, w_0y_ = 14°, λ = 1.31 μm, *l* = 0.1mm, n_eff_ = 3.32, α = 3.5, R_2_ = 0.29 and for a lens of numerical aperture NA = 0.5 and focal length f = 8mm. The mirror reflectivity was taken R_3_ = 0.01.

In this way C becomes dependent on the laser parameters *w*_0_*_y,z_* and λ, and can be corrected by a proper choice of the lens parameters (*f, a*) and of the lens-diode distance *d*. Figure 3 shows the most relevant result of our approach, namely that a careful choice of the beam angular width after the lens allows a compensation of the linear increase of the C-parameter with the target distance x and of the feedback power without any optical filter. C is directly proportional to x as expected from Eq. (1) only for a well - collimated laser beam having d / f ≈ 1, e.g. the case referred in literature as constant mode-matching factor ε.

If d / f < 1, the output beam can be made to diverge and the C-parameter decreases sharply with the mirror distance x until no more filters are required to keep its value within the moderate feedback regime. In this case, only a small fraction of the backward beam re-enters the laser cavity: the greater the laser divergence, the smaller the feedback radiation coupled with the laser [22-23]. Only the fraction of the laser beam orthogonal to the plane target is reflected back following the same optical path toward the collimation lens and contributes to the LSM signal. The remaining part of the laser beam, truncated by the finite aperture of the collimating lens does not contribute to the self-mixing interaction.

## 3. Measurement of yaw and pitch rotations by differential LSM

### 3.1. Basic principle

The measurement technique for the assessment of longitudinal displacements (see Section 2.2) can be easily replicated in order to also evaluate yaw and pitch rotations of the moving target. Each rotation angle of the target can be obtained by simultaneously measuring the linear displacement of three distinct points in the plane of the target. This requires the presence of three identical Self-Mixing Interferometers SMI_i_ (i = 1,2,3), each made of a laser diode L_i_ with integrated monitor photodiode, a lens and a single reflective or retroreflective target M.

**Figure 4.**
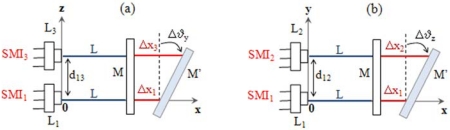
Schematic geometry of the setup for (a) pitch and (b) yaw measurement. Each interferometer SMI_i_ (i = 1,2,3) is made of a laser diode L_i_ and a plane mirror M. M′ is the position of the target after the roto-translation, and L is the distance of the target from the lens before the motion. The monitor photodiode, placed at the rear facet of the laser inside the laser package, and the converging lens have not be drawn in the scheme.

With reference to Figure 4, SMI_1_ and SMI_3_, aligned with the x - axis and placed at a distance of ${\text{d}}_{13}=\bar{{\text{L}}_{1}{\text{L}}_{3}}$ along the z-axis, will measure ϑ_y_ according to Δϑ_y_ = arctan[ (Δx_3_ − Δx_1_)/d_13_]. Analogously, SMI_1_ and SMI_2_, aligned with the x - axis and placed at a distance of ${\text{d}}_{12}=\bar{{\text{L}}_{1}{\text{L}}_{2}}$ along the y-axis, will measure ϑ_z_ according to Δϑ_z_ = arctan[ (Δx_2_ − Δx_1_)/d_12_]. Since the small angle approximation holds throughout the angular range of measurement, the three DOFs are then derived from the following relations:

(7) $$\left\{ \begin{array}{l} \Delta \text{X}={\text{N}}_{1}\cdot \lambda_{1}/2\\ \Delta \vartheta_{\text{z}}≅\left[{\text{N}}_{2}\cdot \lambda_{2}-{\text{N}}_{1}\cdot \lambda_{1}\right]/\left[2{\text{d}}_{12}\right]\\ \Delta \vartheta_{\text{y}}≅\left[{\text{N}}_{3}\cdot \lambda_{3}-{\text{N}}_{1}\cdot \lambda_{1}\right]/\left[2{\text{d}}_{13}\right] \end{array}\right.$$

**Figure 5.**
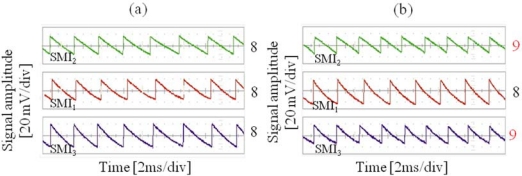
Representative oscilloscope LSM waveforms of the interferometers SMI_i_ (i = 1,2,3), obtained at a distance laser-target of 20 cm for (a) pure translational motion, and (b) translation with both yaw and pitch rotations. The number N of interference fringes is reported to the right of each waveform.

The working principle is demonstrated by inspection of the oscilloscope waveforms reported in Figure 5, where the combined pitch and yaw rotation [Figure 5(b)] results in a different number of interference fringes counted by the three detectors with respect to the pure translational motion [Figure 5(a)].

### 3.2. Experimental setup

**Figure 6.**
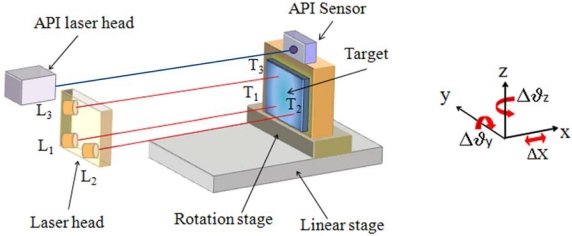
Schematics of the sensor system for 3 DOFs – linear displacement, yaw and pitch – measurement.

The prototype of the sensor is schematically illustrated in Figure 6. It is composed of a laser head, which consists of three parallel laser diodes mounted side-by-side in a “L”-like configuration, and a target made of a plane squared mirror of 70 mm side. The sources are Distributed-Feedback (DFB) laser diodes, with nominal wavelength of 1310 nm, biased at a current I = (23.00 ± 0.01) mA, and equipped with a monitor photodiode integrated into the laser package and an AR-coated aspheric lens.

The photocurrent produced by the detector is firstly AC-coupled to the trans-impedance amplifier of gain = 10^5^ V/A, and then fed into a signal processing board interfaced to a computer for the fringe count. In order to avoid the presence of a variable attenuator, the moderate feedback condition has been achieved by properly defocusing the laser source, following the principle introduced in Section 2.3.

The target is fixed onto a rotation stage, mounted itself onto a 1-meter long linear stage. The minimum distance between the target and the laser head is 20 cm. A commercial 6-axis measurement system (API 6D Laser) has been used as the reference meter. Its nominal resolution is 0.02 μm for longitudinal displacement and 3 × 10^-5^° for angular rotations.

### 3.3. Experimental results

For validating the proposed system as linear displacement sensor, the linear stage was moved along the x-axis in the range -10^3^ – 10^3^ mm at a speed of 10 mm/s. Then, the system was tested as a rotation sensor by keeping the target at constant positions (120 cm from the laser head) and moving the rotation platform by increasing the yaw or pitch angles in both clockwise (-) or anti-clockwise (+) directions in the range ± 0.45°.

**Figure 7.**
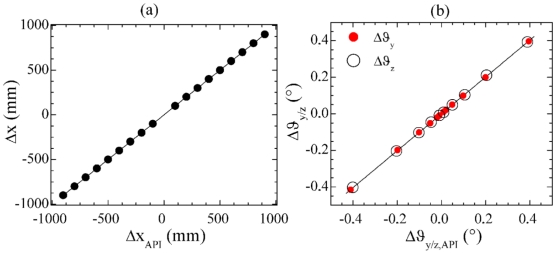
(a) Measured longitudinal displacement Δx as a function of purely linear displacement Δx_API_ measured by the reference system. Negative (positive) values refers to backward (forward) motion with respect to the laser source. (b) Measured yaw (hollow symbols) and pitch (full symbols) rotations as a function of the rotation angle measured by the reference system at a fixed target distance of 120 cm. The vertical error bars are within the symbol size, and the best fit lines, represented by continuous lines, have equations Δx = 0.99999 Δx_API_ - 0.00002 (R^2^ = 1) and Δϑ_y/z_ = 0.9945Δϑ_y/z,API_ + 0.0006 (R^2^ = 0.9999), respectively.

Figure 7 shows the measured linear displacement Δx [Figure 7(a)] and rotations [Figure 7(b)] as compared with the reference values simultaneously measured by the API system. The system response is linear over a longitudinal dynamic range of six orders of magnitude and an angular dynamic range of three orders of magnitude. The maximum continuous linear and angular displacements were only limited by the length of the linear stage and the reference standards.

Improvements in the angular measurement range can be achieved by using three solid retroreflectors (CC), one for each laser, in place of the plane mirror (PT), since in this case the backward optical path is not affected by the target orientation [24].

### 3.4. Comparison with laboratory/commercial existing systems

The only reported measurement of the tilt angle of a remote target via LSM technology was realized by Giuliani *et al*. [25], who exploited the parabolic dependence of the DC power from the tilt angle when the laser diode was driven in the coherence-collapse (high feedback) regime. The technique shows a good sensitivity (0.1 arcsec) but a small angular range (0.06°), and requires the target sitting at a fixed distance from the laser. At the contrary, the proposed three DOFs sensor presents several benefits which can be summarized as follows:

1. Operations in moderate feedback regime allow avoiding the signal instabilities typical of the coherence collapse and, above all, make possible a digital treatment of the signal in place of the analogical signal processing exploited in [25].
2. In spite of a worse resolution, a larger angular measurement range (0.5° in place of the 0.06°) is achieved. On the other side, the system resolution can be improved by increasing the distance between the laser sources.
3. The angular measurement can be performed simultaneously with long linear displacements.

Additionally, a comparison with commercially available instruments employed for industrial metrological applications shows that the demonstrated sensor would fill the existing gap between the sophisticated and costly multi DOFs instruments capable of nanometric resolution and the off-the-shelf instruments capable of sub-millimetric resolution. The intermediate micrometric resolution range is today covered by one DOF linear optical encoders which are unable to handle target rotations. Besides, encoders make use of an “active” reading head with cables and electro-optical components, whose installation on machine tools must satisfy several mechanical constraints. At the contrary, the target of the LSM sensor is “passive”, thus providing a more robust system and a greater flexibility in the sensor integration on machine tool without significant design constraints. These features make the LSM sensor suitable for industrial applications as machine tool calibration and diagnostic.

## 4. Measurement of transverse DOFs

### 4.1. Issues related with the assessment of transverse DOFs: classical and innovative solutions

Every interferometer (both classic or LSM - based) is basically insensitive to any transverse displacement, since the spatial phase **k**·**Δr** - where **Δr** is the displacement vector and **k** the wave vector – is null and thus no interference fringe is produced. Two possible solutions can be adopted to overcome this drawback:

1. The projection of a component of the transverse motion along the direction of the laser beam, via additional optical elements along the light path or specific geometrical configurations.
2. The tilt of the laser beam with respect to the x-axis, so that both linear and transverse displacements have a projection on the new tilted optical axis.

The first solution, commonly explored in literature, is usually achieved by inserting a Wollaston prism on the movable target [26]. When the Wollaston prism is moved along with the stage, a difference in the optical path occurs and the number of interferometric fringes detected by an external receiver is used to measure the straightness of the stage. The measurement resolution is typically some micrometers, and can be improved via the insertion of additional optical elements [27-28]. However, this approach requires expensive setups and cannot be easily extended in a multi – DOF measuring system because of the presence of a fixed element beyond the movable stage, which strictly limits the spatial optimization of the apparatus.

At the opposite, the second approach allows an easy integration with the LSM scheme and only requires a single reference plane - that defined by the laser sources – since the signal detection via the monitor photodiode allows the integration of source and detection elements into a single housing.

### 4.2. Basic principle for straightness and flatness measurements by LSM

**Figure 8.**
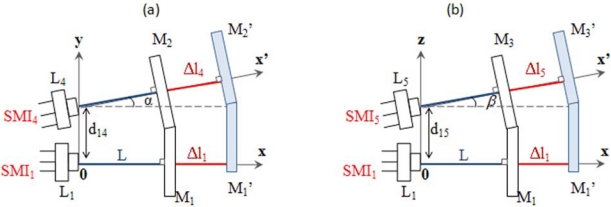
Schematic geometry of the setup for (a) straightness and (b) flatness measurement. Each interferometer SMI_i_ (i = 1,4,5) is made of a laser diode L_i_, and a plane mirror M as target. M′ is the position of the target after the displacement.

With reference to the two SMIs illustrated in Figure 8(a), SMI_1_ is able to detect purely linear displacement Δx along the x-axis whereas it is totally blind to any transverse motion. A second interferometer SMI_4_, rigidly tilted by a small angle α with respect to the x-axis in the xy plane, identifies a new optical axis x'. This expedient makes the interferometer sensitive to displacements along both the linear (x) and transverse (y) axis, since both cause a change Δl_4_ = ± N_4_ × λ_4_ /2 of the optical path length along x'.

Only SMI_4_ is thus needed for measuring a pure Δy displacement, whereas a combined linear / transverse displacement requires the comparison of the two SMIs readings in order to be properly measured according to:

(8) $$\left\{ \begin{array}{l} \Delta \text{x}=\Delta {\text{l}}_{1}\\ \Delta \text{y}=\Delta {\text{l}}_{4}/\sin\alpha -\Delta {\text{l}}_{1}/\tan(\alpha ) \end{array}\right.$$

The same considerations apply for the measurement of flatness, via a third interferometer SMI_5_, tilted by an angle β with respect to the x – axis in the xz plane [see Figure 8(b]. In terms of number of interference fringes, the measurement displacements can be written as:

(9) $$\left\{ \begin{array}{l} \Delta \text{x}={\text{N}}_{1}\cdot \lambda_{1}/2\;\\ \Delta \text{y}={\text{N}}_{4}\cdot \lambda_{4}/\left[2\sin(\alpha )\right]-{\text{N}}_{1}\cdot \lambda_{1}/\left[2\tan(\alpha )\right]\\ \Delta \text{z}={\text{N}}_{5}\cdot \lambda_{5}/\left[2\sin(\text{β})\right]-{\text{N}}_{1}\cdot \lambda_{1}/\left[2\tan(\text{β})\right] \end{array}\right.$$

Similarities with the measurement technique for yaw and pitch rotations are remarkable: straightness and flatness displacements can be obtained by three identical self-mixing interferometers SMI_i_ (i = 1,4,5), via the simultaneous measurement of the linear displacement of three distinct points on a specially designed target.

### 4.3. Basic principle for roll measurements

Two preliminary considerations may help the introduction of our solution for the DOF most elusive to interference. First, roll ϑ_x_, that is the rotation along the x - axis, can be basically measured similarly to the yaw or pitch rotation (see Paragraph 3.1), i.e. by means of a differential technique by two parallel interferometers SMI_i_ and SMI_j_. This means Δϑ_x_ &prop; (Δl_i_ − Δl_j_)/d_ij_. Second, roll ϑ_x_ is a transverse angular displacement, thus requiring the tilt of the two laser beams with respect to the x-axis as for the straightness and flatness (see Paragraph 4.2). This means Δϑ_x_ ≈ [Δl_i_ − Δl_j_]/ [d_ij_·sin(ξ)], with ξ the tilt angle of the mirror employed in the roll measurement, i.e. ξ = α or β in case of M_2_ or M_3_, respectively.

One can exploit, at this aim, the laser source L_5_ already used for flatness measurement, and a new laser diode L_6_, both pointed against the mirror M_3_ and separated along the y-axis by a distance d_56_. A pure roll rotation can thus be derived from the differential readings of the two SMI_i_ (i = 5,6) by means of the following expression Δϑ_x_ ≈ [Δl_6_ − Δl_5_]/ [d_56_·sin(β)] or, in terms of number of fringes, as:

(10) $$\Delta \vartheta_{\text{x}}≅\left[{\text{N}}_{6}\cdot \lambda_{6}-{\text{N}}_{4}\cdot \lambda_{4}\right]/\left[2\cdot {\text{d}}_{46}\cdot \sin\left(\alpha \right)\right]$$

### 4.4 Experimental setup and results

**Figure 9.**
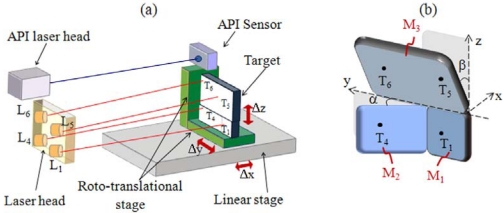
(a) Schematics of the setup for a transverse measuring system. (b) Details of the target.

The prototype of the sensor, illustrated in Figure 9 (a), is similar to that previously described in Figure 6. The only difference consists in the target configuration, which is given by three reciprocally tilted mirrors [Figure 9(b)]: M_1_ is a squared mirror of side 20 mm lying in the plane yz, M_2_ is a rectangular mirror of dimension 50 mm × 20 mm, tilted at angle α = (2.1 ± 0.1)° around the z – axis, M_3_ is a rectangular mirror (70 mm × 50 mm) tilted at angle β = (1.8 ± 0.1)° around the y - axis.

**Figure 10.**
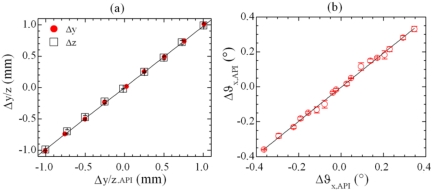
(a) Transverse target displacement Δy (red marks) / Δz (black marks) and (b) roll Δϑ_x_, measured at a distance of 120 cm from the laser source, as a function of the reference displacement, whose nominal resolutions are 0.1 μm for transverse displacement and 3 × 10^-5^° for roll rotations. The vertical error bars are nearly always within the symbol size. The best linear fit, represented by the continuous lines, have equations Δy/z = 1.02197 Δy/z_API_ + 0.00155 (R^2^ = 0.9997) and *Δϑ_x_* = 0.9768Δϑ_x,API_ - 0.00287 (R^2^ = 0.9961).

To validate the proposed system as a transverse displacement sensor, the interferometers SMI_i_ (i = 4-6) were tested for displacements Δy and Δz in the range ± 1 mm [Figure 10(a)] and for rotation Δϑ_x_ in the range ± 0.4° [Figure 10(b)], at a fixed distance laser/ target L = 120 cm. The linearity of the response is guaranteed over the full measurement range, that is mainly limited by the available stages and reference meter ranges.

## 5. System performances: measurement resolution and accuracy

Given the intrinsic linear resolution λ/2 of the LSM technique, the resolution of the other 5 DOFs can be derived by equations (7-10) assuming null one of the counters and unitary the other, resulting in:

(11) $$\left\{ \begin{array}{l} {\text{R}}_{\text{x}}=\lambda /2=0.7\mu \text{m}\\ {\text{R}}_{\text{y}/\text{z}}≅\lambda /\left[2\cdot \sin(\alpha )\right]≅18\mu \text{m}\\ {\text{R}}_{\vartheta \text{z}/\text{y}}≅\lambda /\left[2\cdot {\text{d}}_{13}\right]≅10^{-3}{}^{\circ}\\ {\text{R}}_{\vartheta \text{x}}≅\lambda /\left[2\cdot {\text{d}}_{56}\cdot \sin(\alpha )\right]=1.5\times 10^{-2}{}^{\circ} \end{array}\right.$$

where:

- the wavelengths of the six lasers are assumed comparable and equal to λ = 1.3 μm;
- the distances between the laser diodes were d_12_ ≈ d_13_ = (50.0 ± 0.1) mm and d_56_ = (66.0 ± 0.1) mm;
- the tilt angles of the mirrors M_i_ (i=2,3) are equal to α ≈ β = (2.1 ± 0.1)°.

The estimated measurement accuracy σ, that is the deviation between the measured and the actual displacement, is obtained by the error propagation formula as:

(12) $$\left\{ \begin{array}{l} \sigma_{\text{x}}={\left[{\left(\frac{\lambda }{2}\right)}^{2}{\sigma_{\text{N}}}^{2}+\Delta {\text{x}}^{2}{\left(\frac{\sigma_{\lambda }}{\lambda }\right)}^{2}\right]}^{1/2}\\ \sigma_{\text{z}}\approx \sigma_{\text{y}}={\left[2{\left(\frac{\sigma_{\text{x}}}{\alpha }\right)}^{2}+\Delta {\text{y}}^{2}{\left(\frac{\sigma_{\alpha }}{\alpha }\right)}^{2}\right]}^{1/2}\\ \sigma_{\vartheta \text{z}}\approx \sigma_{\vartheta \text{y}}={\left[2{\left(\frac{\sigma_{\text{x}}}{{\text{d}}_{13}}\right)}^{2}+{\Delta \vartheta_{\text{y}}}^{2}{\left(\frac{\sigma_{{\text{d}}_{13}}}{{\text{d}}_{13}}\right)}^{2}\right]}^{1/2}\\ \sigma_{\vartheta \text{x}}={\left[2{\left(\frac{\sigma_{\text{x}}}{{\text{d}}_{56}\alpha }\right)}^{2}+{\Delta \vartheta_{\text{x}}}^{2}{\left(\frac{\sigma_{{\text{d}}_{56}}}{{\text{d}}_{56}}\right)}^{2}+{\Delta \vartheta_{\text{x}}}^{2}{\left(\frac{\sigma_{\alpha }}{\alpha }\right)}^{2}\right]}^{1/2} \end{array}\right.$$

where the small angle approximation has been made and the individual terms are:

- the error σ_N_ about the total fringe count N = N_+_ - N_-_, due to the precision of the counting system. It is assumed σ_N_ = 2 due to the intrinsic uncertainty of ± 1 count for each electronic channel (uncompleted fringe);
- the error σ_λ_ about the laser wavelength. This error has been obtained by continuously monitoring the wavelength with a wavelength meter allowing a relative accuracy σ_λ_/λ = 10^-5^. However, analogous results can be realized via both the active temperature stabilization of the diode temperature to 0.01 °C or a temperature-calibrated DFB laser source [29];
- the error σ_d_ about the distance between the diode lasers. By calibrating the LSM sensor against a reference angular meter, the distances d_ij_ can be evaluated as fitting parameters with an error of σ_d_ = 0.1 mm;
- the error σ_α,β_ on the tilt angle between the diode lasers. After a preliminary calibrations, the tilt angle α ≈ β can be assessed as fitting parameters with an uncertainty of σ_α,β_ = 0.1°.

The estimated accuracy for the maximum linear and angular displacement considered individually in the previous experiments, is σ_x_ = 11 μm for a longitudinal displacement Δx = 1 m, σ_y_ = σ_z_ = 70 μm for a transverse displacement Δy = Δz = 1 mm, σ_ϑy_ = σ_ϑz_ = 3 × 10^-3^° for yaw or pitch rotations Δϑ_y_ = Δϑ_z_ = 0.5 °, and σ_ϑx_ = 5 × 10^-2^° for roll rotations Δϑ_x_ = 0.5 °.

Equations (11,12) show that the performance of the system scales with the spatial separation d_ij_ between the laser diodes as concern the rotations, and the tilt angles α/β of the reflective surfaces as concern the transverse motion. Both these parameters directly affects the system dimension and can be easily improved by using a larger target and a bulkier laser head, thus requiring a delicate trade – off between performance and compactness of the sensor.

For example, the angular resolution can be halved by doubling the distance d_ij_ between the lasers L_i_ and L_j_. On the other side, a straightness resolution of 1 μm, comparable with the longitudinal displacement, can be achieved with a wavelength λ of 0.8 μm and an angle α approximately equal to 25°. However, such a large angle is not practical if medium – long linear displacements (some centimetres up to some meters) are allowed along the x – axis, since the drift of the spot of the tilted laser L_4/5_ in the yz plane would increase proportionally to Δx. Accordingly, the system resolution will be constrained by the dimensions of the system: given the maximum allowed size of the target (h × h) and its maximum longitudinal distance from the lasers L_max_, the tilt angle must satisfy the following condition:

(13) $$\alpha \leq \arctan\left[\text{h}/{\text{L}}_{\max}\right]$$

**Figure 11.**
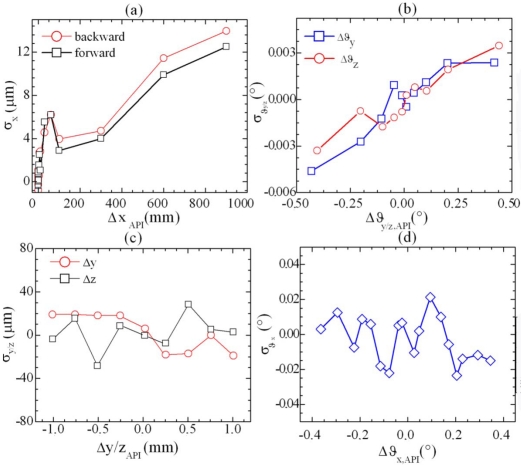
Measurement error for (a) linear displacement, (b) yaw and pitch rotations, (c) transverse displacement, and (d) roll rotations. The theoretical accuracy estimated for the maximum displacements performed is approximately 11 μm for linear displacement, ± 70 μm for transverse displacement, ± 3 × 10^-3^° for yaw and pitch, and ± 5 × 10^-2^° for roll.

The errors for each DOF, evaluated as the difference between the values measured by the SMIs and those given by the reference meter, are reported in Figure 11 and are within the theoretical accuracy estimated by means of equation (12).

## 6. Simultaneous assessment of more DOFs

### 6.1. Experimental results

**Figure 12.**
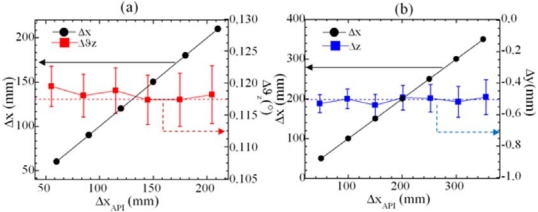
(a) Simultaneous measurement of a linear displacement in the range 50 – 220 mm with a fixed yaw rotation Δϑ_z_ = 0.117°, pointed out by the red dotted line. The best fit line equation related to the linear displacement is Δx = 1.00000 Δx_API_ + 0.00695 (R^2^ = 1) (b) Simultaneous measurement of a linear displacement in the range 20 – 400 mm with a fixed straightness Δy = - 0.5 mm, pointed out by the blue dotted line. The best fit line equation related to the linear displacement is Δx = 1.00001 Δx_API_ - 0.00311 (R^2^ = 1). Arrows in (a) and (b) point to the relevant axis for the corresponding set of data.

Most practical applications require a real-time control of small five DOFs deviations of a moving target while it is moving along the main x-axis. Interestingly, the proposed technique allows the simultaneous measurement of more DOFs, as demonstrated in Figure 12 where a linear increasing translations Δx is imposed to the target together with a fixed yaw rotation Δϑ_z_ of about 0.12° (Figure 12 a) or a fixed straightness displacement of Δy = - 0.5 mm [Figure 12(b)]. The results shows that the combination of linear and angular motion can be handled consistently by SMIs without a significant loss of accuracy.

### 6.2. Mathematical algorithm

In the previous sections, we reported on the profiling of the LSM technique for the measurement of the six DOFs by using an intuitive geometrical approach for each DOF. Nonetheless, identical results can be achieved in an analytical frame by means of a unique mathematical algorithm able to convert the six counts of fringes N_i_ (i=1…6) into the direct measurement of linear and angular displacements of the target, and vice-versa. This kind of analysis becomes necessary in presence of random displacement of the target, which can present simultaneously up to six DOFs and thus requires a re-definition of the measurement expressions for taking into account the mutual interactions between the different DOFs.

**Figure 13.**
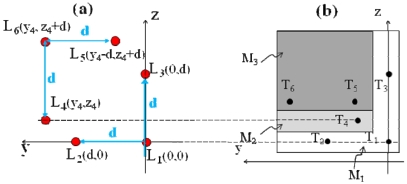
(a) Possible arrangement of the six laser sources L_i_ and (b) of the corresponding target configuration in a 6 DOFs sensor. Dimensions are d = 50 mm, (y_4_,z_4_) = (65,15) mm, M_1_ = 70×70 mm^2^, M_2_ = 10×60 mm^2^, M_3_ = 60×50 mm^2^. T_i_ (i = 1,2,3) indicate the fixed spots of L_i_ (i = 1,2,3). T_i_ (i = 4,5,6) indicate the point of incidence of L_i_ (i = 4,5,6) when the target is at the maximum distance x_max_. For x < x_max_ T_4_ moves horizontally away from the z-axis and T_5,6_ move vertically away from the y-axis.

Figure 13 reports a possible configuration of a unique target for a 6-DOFs self-mixing system, chosen in order to optimize the geometry and the compactness of the system. The target is composed by a squared mirror M_1_ lying in the yz plane for the measurement of linear displacement, yaw and pitch, a rectangular mirror M_2_ tilted by a yaw α for the measurement of straightness, and a second rectangular mirror M_3_ tilted by a pitch β for the measurement of flatness and roll.

The main steps in the development of the algorithm are reported in the following. First, the relative change of the optical path Δδ_ij_ = δ_ij_ − δ_ij,0_ between the laser source L_i_ and the target M_j_ is provided by Δδ_ij_ = N_i_ · λ_i_, where δ_ij_ and δ_ij,0_ are the optical path at the end and at the start of the motion, respectively, and the net count of interference fringes N_i_ produced during the motion only depends by the initial and final position assumed by the target.

Second, the optimal target for 6-DOFs measurement is assumed to be composed of three planes with no reciprocal symmetry. The lack of symmetry is validated when there is a single intersection point between the three planes M_j_ or, analytically, when their normal versors r̂_j_ r̂_j_ are linearly independent. Thereinafter, M_1_ will be used for linear displacement (i = 1,2,3, j = 1), M_2_ for straightness (i = 4, j = 2) and M_3_ for flatness and roll (i = 5,6, j = 3), so that the couple of indexes (i,j) identify the six interferometric channels SMI_ij_, each composed by a laser head and a plane target.

Third, in the LSM scheme it will be assumed that δ_ij_ agrees with twice (considering the forward and backward path) the geometrical distance between the point-like laser source and the plane of the target, since the relevant optical path in the LSM scheme is given by the ray orthogonal to the mirror, reflected backward following the same parallel direction. In a fixed right-handed reference frame, we have:

(14) $$\Delta \delta_{\text{ij}}=2({\vec{\text{t}}}_{\text{i}}-{\vec{\text{d}}}_{\text{i}})\cdot {\hat{\text{r}}}_{\text{j}}$$

where the vector $\vec{{\text{d}}_{\text{i}}}$ (i = 1..6; six laser sources) defines the position of the laser source L_i_ pointed against the target M_j_, the vector $\vec{{\text{t}}_{\text{j}}}$ (j = 1..3; three reflective targets) defines the position of a generic point T_j_ of the mirror M_j_ whose normal versor is r̂_j_. The change in the optical path can then be obtained as:

(15) $${\text{N}}_{\text{i}}\cdot \lambda_{i}/2=({\vec{\text{t}}}_{\text{j}}-{\vec{\text{d}}}_{\text{i}})\cdot {\hat{\text{r}}}_{\text{j}}-(\vec{{\text{t}}_{\text{j}0}}-\vec{{\text{d}}_{\text{i}}})\cdot {\hat{\text{r}}}_{\text{j0}}$$

A simplification of equation (15) can be introduced replacing the three vectors $\vec{{\text{t}}_{\text{j}}}$ by a single vector t⃗ which defines the position of the intersection point T between the three planes of the mirrors. The existence and the uniqueness of the intersection point is derived from the linear independency of the versors r̂_j_. Thereby, equation (15) can be re-written as:

(16) $${\text{N}}_{\text{i}}\cdot \lambda_{\text{i}}/2=(\vec{\text{t}}-{\vec{\text{d}}}_{\text{i}})\cdot {\hat{\text{r}}}_{\text{j}}-(\vec{{\text{t}}_{0}}-\vec{{\text{d}}_{\text{i}}})\cdot {\hat{\text{r}}}_{\text{j0}}$$

where the wavelengths λ_i_, the initial linear position $\vec{{\text{t}}_{0}}$ and the initial angular position r̂_j0_ of the target, and the laser positions $\vec{{\text{d}}_{\text{i}}}$ are exactly known.

Equation (16) is the core of the LSM analytical approach, and can be exploited in two complementary ways:

1. to recover the 6 counts N_i_ once known the final target position t⃗_\_and orientation r̂_j_. In this case equation (16) represents a parametric system of 6 equations (i = 1..6) in 6 variables, which can be exactly solved;
2. to recover the final target position t⃗ and orientation r̂_j_, once known the counts N_i_. In this case equation (16) represents a parametric system of 6 equations (i = 1..6) in 12 scalar variables (4 variables, each having three Cartesian components x,y,z). The remaining six equations required to solve the system are provided by the 3 normalization conditions ${\left[{\hat{\text{r}}}_{\text{jx}}^{2}+{\hat{\text{r}}}_{\text{jy}}^{2}+{\hat{\text{r}}}_{\text{jz}}^{2}\right]}^{1/2}=1$ on the versors r̂_j_ and the 3 equations describing their mutual orientation. In this way, a solvable system of 12 equations (i = 1..6) in 12 scalar variables can be obtained. The analytical complexity of such a system, often preventing it from being solved, is significantly reduced in presence of purely translational or rotational displacements.

For example, in presence of pure longitudinal and transverse displacements, the versors r̂_j_ describing the angular orientation of the target can be assumed constant during the motion, so that r̂_j_ = r̂_j0_. As a result, equation (16) simplifies to:

(17) $${\text{N}}_{\text{i}}\cdot \lambda_{\text{i}}/2=(\vec{\text{t}}-{\vec{\text{t}}}_{0})\cdot {\hat{\text{r}}}_{\text{j}0}$$

By making explicit the index *i* = 1,4,5 of the lasers involved in the measurement of translations, and by writing the normal versors r̂_j0_ [see Figure 9(b)] as:

(18) $${\hat{\text{r}}}_{10}=(1,0,0);{\hat{\text{r}}}_{20}=(\cos\alpha ,\sin\alpha ,0);{\hat{\text{r}}}_{30}=(\cos\beta ,0,-\sin\beta )$$

the indexed equation (17) gives rise to the following system:

(19) $$\left\{ \begin{array}{l} {\text{N}}_{1}\cdot \lambda_{1}/2=\Delta \text{x}\\ {\text{N}}_{4}\cdot \lambda_{4}/2=\Delta \text{x}\cdot \cos(\alpha )+\Delta \text{y}\cdot \sin(\alpha )\\ {\text{N}}_{5}\cdot \lambda_{5}/2=\Delta \text{x}\cdot \cos(\text{β})-\Delta \text{z}\cdot \sin(\text{β}) \end{array}\right.$$

Equation (19) allows retrieving the translational DOFs by means of a system analogous to equation (7), except for a minus sign in the flatness Δz coming from a correct consideration of the angle sign in the right-handed reference frame.

A similar procedure provides the estimation of rotational DOFs which, as an intermediate step, requires the knowledge of the rotated versors r̂_j_ from the global rotation matrix, given as the right- product of the yaw/pitch/roll rotations matrices. Details can be found in [23].

## 7. Conclusions

In this paper, we have reached and surpassed the state-of-the art performance in the measurement of linear displacement of a reflective target, by demonstrating a continuous longitudinal dynamic range up to 2 meters with sub-micrometric resolution and without any external optical attenuator along the external cavity.

The increased useful range of LSM sensor has been achieved by means of a divergent laser beam in place of the typically collimated one, in order to properly adjust the feedback power ratio in such a way to compensate for the linear dependence of the C-parameter with the target distance.

Besides, we have demonstrated for the first time that the LSM effect can be used to simultaneously measure the 6 DOFs (three translations and three rotations) of a remote target. This result has been achieved by the differential measurement of couples of identical LSM interferometers, properly aligned with respect to the longitudinal axis in order to recover both the linear and the transverse information.

The proof of principle of each DOF measurement has been demonstrated by implementing a prototype experimental setup, made of DFB laser sources and an innovative target consisting of three reciprocally tilted mirrors attached to the moving object. The performance improves proportionally to the laser wavelength and is constrained by the dimension of the target and the length of the linear track. In the present configuration, a resolution of ± 0.7 μm for longitudinal translations over ± 1 m, ± 20 μm for transverse translations over ± 1 mm, ± 10^-3^ ° (yaw and pitch) and ± 1.5 × 10^-2^ ° (roll) for rotations over ± 0.45°, has been achieved.

In spite of a comparable resolution and accuracy with respect to the traditional opto-mechanical systems, the proposed LSM sensor is inherently compact, easy to align, and provide direct measurements of displacement by the digital count of the number of interference fringes, with no need of interpolation. Hence, it is suitable for several industrial applications, especially in mechatronics and robotics, where machine-tool calibration or motion monitoring systems are highly desirable.
